# Postoperative IMRT in head and neck cancer

**DOI:** 10.1186/1748-717X-1-40

**Published:** 2006-10-19

**Authors:** Gabriela Studer, Katrin Furrer, Bernard J Davis, Sandro S Stoeckli, Roger A Zwahlen, Urs M Luetolf, Christoph Glanzmann

**Affiliations:** 1 Radiation Oncology, University Hospital Zurich, Rämistrasse 100, 8091 Zurich, Switzerland; 2 Otorhinolaryngology, Head and Neck Surgery, University Hospital Zurich, Frauenklinikstrasse 24, 8091 Zurich, Switzerland; 3 Department of Cranio-Maxillofacial Surgery, University Hospital, Zurich, Switzerland

## Abstract

**Background:**

Aim of this work was to assess loco-regional disease control in head and neck cancer (HNC) patients treated with postoperative intensity modulated radiation therapy (pIMRT). For comparative purposes, risk features of our series have been analysed with respect to histopathologic adverse factors. Results were compared with an own historic conventional radiation (3DCRT) series, and with 3DCRT and pIMRT data from other centres.

Between January 2002 and August 2006, 71 patients were consecutively treated with pIMRT for a squamous cell carcinoma (SCC) of the oropharynx (32), oral cavity (22), hypopharynx (7), larynx (6), paranasal sinus (3), and an unknown primary, respectively. Mean and median follow up was 19 months (2–48), and 17.6 months. 83% were treated with IMRT-chemotherapy. Mean prescribed dose was 66.3 Gy (60–70), delivered with doses per fraction of 2–2.3 Gy, respectively.

**Results:**

2-year local, nodal, and distant control rates were 95%, 91%, and 96%, disease free and overall survival 90% and 83%, respectively. The corresponding survival rates for the subgroup of patients with a follow up time >12 months (n = 43) were 98%, 95%, 98%, 93%, and 88%, respectively. Distribution according to histopathologic risk features revealed 15% and 85% patients with intermediate and high risk, respectively. All loco-regional events occurred in the high risk subgroup.

**Conclusion:**

Surgery followed by postoperative IMRT in patients with substantial risk for recurrence resulted in high loco-regional tumor control rates compared with large prospective 3DCRT trials.

## Background

Despite high dose postoperative irradiation in patients with locally advanced head and neck cancer (HNC) with certain high risk factors, loco-regional recurrences occur in approximately 30% of the cases [1,2]. Three randomized studies showed an increase of loco-regional control and overall survival when postoperative radiation and concomitant chemotherapy are combined [2-4] (Table 1). Accelerating treatment by using concomitant boost did not result in a clear improvement of loco-regional control [5] except perhaps in patients with a longer interval between surgery and radiation. In our own experience in postoperative radiation using concomitant boost, local control was 83% in accelerated vs 68% with conventional fractionation (S Maurer, dissertation, Radiation Oncology, University Hospital of Zurich, 1996). Since approximately 5 years, intensity modulated radiation therapy (IMRT) has been introduced in the clinic and used in HNC. This has resulted in a high loco-regional control [6-8] and better tolerance [6], compared to the "traditional 3DCRT".

**Table 1:** Comparison with historic conventional postoperative three-dimensional radiation therapy (p3DCRT) data

			**2-year control rates**						
							
**Author **reference)	**n**	**risk level**	**% LRC**	**% DFS**	**% OAS**	***% *high risk**	**pRT technique**	**pRT dose**	**cc ChTh**
**Aug et al **[5]	151	HR	~72	na	~60	100%	3dcrt	63 Gy	no
	62	LR, IR	~95	na	~90	0%	3dcrt	LR: no RT, IR: 57.6 Gy	no
**Cooper et al **[4]	228	HR	~83	~55	~65	100%	3dcrt	60–66 Gy in 30–33 f	yes
	231	HR	~72	~50	~55	100%	3dcrt	60–66 Gy in 30–33 f	no
**Bernier et al **[3]	167	HR	~82	~67	~75	> 60%	3dcrt	66 Gy in 33 f	yes
	167	HR	~70	~48	~61	> 60%	3dcrt	66 Gy in 33 f	no
**Bachaud et al **[2]	39	HR	79	65	75	100%	3dcrt	NA	yes
	44	HR	59	41	44	100%	3dcrt	NA	no
**Porceddu et al **[13]	47	HR	~73	~56	~62	100%	3dcrt	mean 60 Gy (50–66)	yes
**own**	60	HR	92	90	81	100%	IMRT	60–70 Gy in 27–35 f	most (>80%)
	11	IR	100	90	90	0%	IMRT	60–70 Gy in 27–35 f	most (>80%)

Comparison with historic conventional postoperative three-dimensional radiation therapy (p3DCRT) data

We are presently assessing the results focussing on the validity of anatomic target definitions and the dosage, respectively.

IMRT data on postoperative cohorts are still scant [9-12] (Table 2). Risk feature assessment has not been discussed in the published data.

**Table 2:** 

**Author, **ref	**(year)**	**n pIMRT (dIMRT)**	**HNC subsites**	**T3/4, rec, OCC**	**pIMRT dose**	**Chemotherapy**	**2-y L(R)C**	**median FU**
**Lee et al **[15]	**(2003)**	43 (107)	all	53, 0, 2%	66 Gy	35% of pIMRT	83% LC	25 (6–78)
**Chao et al **[9]	**(2004)**	74 (52)	all	52, 0, 12%	~68 Gy (+/-4.7)	none of pIMRT	90% LRC	26 (12–55)
**Feng et al **[10]	**(2005)**	86 (72)	all but NPC/SNC	90 lll/lV, 3, 23%	~70 Gy (66–76)	12% of all	~85% LRC	36 (6–127)
**Yao et al **[12]	**(2005)**	51 (100)	all	53, 0, 19%	64–66 Gy	none of pIMRT	~92% LC	18 (2–60)
**own**	**(2006)**	71 (230)	all SCC	25, 18, 31%	~66 Gy (60–70)	83% of pIMRT	95% LC	17.6 (2–48)
							91% NC	

Published postoperative IMRT (pIMRT) results in head and neck cancer (HNC)

(dIMRT: number of patients treated with definitive IMRT, rec: recurrence, OCC: oral cavity cancer, FU: follow up)

In order to compare our own data with published results, outcome parameters and risk factors as established by Ang et al [5], have been used.

## Results

Patient and tumor characteristics are listed in Table 3. Postoperative HNC patients treated in the same time interval for histopathologic diagnosis other than squamous cell carcinoma (n = 15) have been excluded from this analysis.

**Table 3:** 

**factors**	**n**
**gender**	61 m : 10 f
**age**	59 (38–85)
**diagnosis**	
**oropharynx**	32
**oral cavity**	22
**hypopharynx**	7
**sinus**	3
**larynx**	6
**unknown, N+**	1
**T stages**	
Tx	1
T1	18
T2	21
T3	6
T4	12
recurrence *	13
**N stages**	
N0	15
N1	4
N2a-b	43
N2c	6
N3	3
concomitant CT **	59 (83%)
**median/mean FU**	17.6/19 months

Patient and tumor characteristics in 71 patients treated with postoperative SIB-IMRT

*: recurrence following surgery alone (none of all patients underwent previous irradiation; all recurred lesions have been re-operated prior to postoperative IMRT)

**: Cisplatinum based chemotherapy (in additional two patients with contraindications for cisplatinum, cetuximab has been given in combination with IMRT)

**Figure 1:**
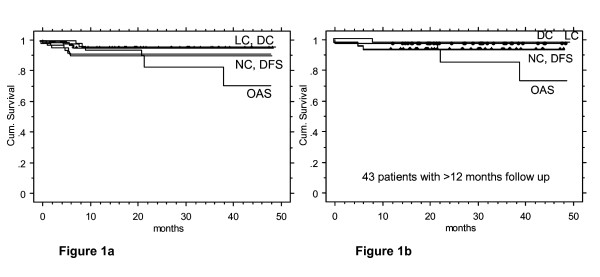
**a: **2-year actuarial local (LC, 95%), nodal (NC, 91%), distant control (DC, 96%), and disease free (DFS, 90%) and overall survival rate (OAS, 83%) in 71 postoperative IMRT patients.**b: **2-year actuarial local (LC, 98%), nodal (NC, 95%), distant control (DC, 98%), and disease free (DFS, 93%) and overall survival rate (OAS, 88%) in 43/71 postoperative IMRT patients with a follow up period of >12 months.

Local, nodal, distant control, disease free and overall survival rates at 2 years were 95, 91, 96%, 90% and 83%, respectively, for the entire cohort (Figure 1a). The corresponding survival rates for the subgroup of patients with a follow up time >12 months (n = 43) were 98%, 95%, 98%, 93%, and 88%, respectively (Figure 1b).

The small number of events did not allow further subgroup analysis with respect to diagnosis, age or gender.

When last seen, 63 patients (89%) were alive with no evidence of disease, one was alive with disease, 3 patients had died of disease, and 4 of independent reasons not related to the tumor.

Staging according to pathologic features established by Ang et al [5] revealed 15% and 85% patients with intermediate and high risk disease, respectively. Table 4 shows the histopathologic risk factors, on which the risk stratification is based.

**Table 4:** 

**Adverse factors**	**n patients [na]**
**ECE nodal**	33 [1]
**>1 nodal group**	30 [2]
**>/= 2 pos LN**	34 [2]
**> 3 cm LN**	19 [4]
**OCC**	22 [0]
**R1**	51 [5]
**PNI**	11 [6]

Histopathologic risk factors (na: not assessable, ECE: extra-capsular extension, LN: lymph node, OCC: oral cavity cancer, R1: microscopically positive margin, PNI: perineural infiltration)

Six patients with 3 local, 5 nodal, and 2 distant relapses were observed. The follow up time is still short. However, considering the fact that 90% of all loco-regional recurrences occurred during the first 12 months after completion of radiation in our definitive IMRT cohort (64 out of 69 local and nodal events in 230 patients), and all events observed in the pIMRT cohort occurred during the first 10 months, the observation interval of median/mean 17.6/19 months (2–48) is expected to be long enough for a representative estimation of the 2-year outcome in pIMRT.

All patients with loco-regional failure belong to the histopathologic high risk group. Two of the three failed patients were referred for resected recurrence of an oral cavity and glottic cancer, respectively. The third locally failed patient suffered from a pT2pN2 oral cavity tumor.

Nodal failure in locally controlled patients occurred in three patients. None of the loco-regionally controlled patients developed metastatic disease.

All nodal failures occurred in nodally dissected patients with proven pN+ disease (ECE in 3/5).

**Figure 2:**
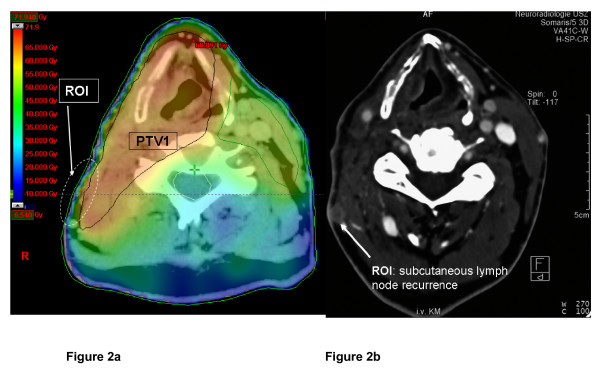
Superficial nodal recurrence (ipsilateral dorsolateral node) in a patient with a pT2 pN2b supraglottic larynx carcinoma. Preoperative diagnostic images did not show any suspicious superficial nodes, nor were any enlarged nodes visible in the postoperative planning computed tomography **a**: IMRT treatment plan, PTV1 (black line): 'build up' effect in the skin/subcutaneous region of interest (ROI, white dotted line) which was not intended to be included into the PTV1. **b**: posttreatment follow up computed tomography scan revealed a superficial lymph node metastasis (ROI), located in the former 'build up' area

The CT of all patients with loco-regional failures were reviewed, and recurred tumor manifestations correlated with the drawn contours and isodoses on the treatment plan. Loco-regional failures were confirmed as 'in-field' relapses (>95% of the tumor volume inside the 95% PTV isodose) in all but one case with a superficial nodal relapse (Figure 2a, b).

### Treatment times

The interval between surgery and the start of radiation was 4–6 weeks (median 36 days) in 11, and >6 weeks (median 45 days) in 60 treated individuals; the pIMRT duration was mean 44 days (37–47, Table 5). Using an slightly accelerated dose per fraction to the SIB target volume (i.e. 2.11, 2.2, 2.3 Gy per fraction), as performed in 24 of the 71 SIB-IMRT cases, an additional mild dose acceleration has been reached.

**Table 5:** 

	**postoperative SIB-IMRT schedules**				
		
**n patients**	**PTV1**	**PTV2**	**PTD PTV1/PTV2 (Gy)**	**fractionation**	**TRT (days)**
**47**	30–35 × 2.0 Gy	30–35 × 1.64–1.8 Gy	60–70/48–56	5/w	38–47
**16**	30–33 × 2.11 Gy	30–33 × 1.64 Gy	63.3–69.6/49.2–54	5/w	40–45
**7**	27–30 × 2.2	27–30 × 1.8 Gy	59.4–66/48.6–54	5/w	37–40
**1**	27 × 2.3 (LN), 2.2 Gy	27 × 1.8 Gy	62.1, 59.4/48.6	5/w	37

Used simultaneously integrated boost (SIB) IMRT schedules in our postoperatively irradiated patients

(PTV1: planning target volume 1 = boost volume, PTV2: planning target volume 2 = elective treatment volume, PTD: prescribed total dose, TRT: total radiation time, 3DCRT: three-dimensional conformal radiation therapy)

#### Toxicity

Acute toxicity was mild to moderate. No grade 4 reactions, and no treatment interruptions occurred due to radiation or chemotherapeutic side effects, respectively. A gastric feeding tube was used in 10 patients (14%), body weight in the entire cohort was mean 71 kg prior to radiation, mean 68 kg at the end of pIMRT, and mean 67 kg at 1 year from completion of radiation, respectively. The maximum individual weight loss during radiation was 9% of the pre-treatment weight.

No grade 4 reaction, and only one grade 3 late effect (xerostomia) was observed in the 43 patients with an at least 12 months follow up period.

## Discussion

Treatment outcome of HNC patients treated with postoperative IMRT has been assessed.

We found a high local-regional disease control rate in a collective of 71 patients of these 60 had high risk features, and 13 were referred for recurrent disease after surgery alone.

Peters et al [1] (1993) and Ang et al [5] (2001) undertook prospective randomized trials to address the validity and dose of postoperative radiation, the impact of accelerating postoperative radiation, and the importance of the overall treatment time on outcome in 302 and 213 patients, respectively. The authors found significantly higher loco-regional control (LRC) and survival rates in non irradiated low risk and irradiated intermediate risk patients compared to high risk patients irradiated with higher doses. In addition, for high risk patients, a trend toward higher LRC was found when radiation was delivered in 5 rather than in 7 weeks, and a significant LRC benefit was shown when the interval between surgery and radiation in the 7-week schedule was short. Consequently, the cumulative duration of combined therapy has a significant impact on LRC and survival.

Concomitant chemotherapy with cisplatin has been investigated in 3 randomised series:

Bachaud et al [2] (1991) showed a significant improvement of LRC as well as survival rates in a relatively small number of patients (n = 83). These results have been confirmed and extended by Cooper et al [4], and Bernier et al [3], respectively. Cooper et al [4] (Table 1) assessed 459 high risk patients enrolled into an intergroup phase lll trial (RTOG, ECOG, SWOG) to test the hypothesis that concurrent postoperative radiation-chemotherapy would improve LRC compared to the radiation alone arm. The difference was significant in favour to the combined approach, with 82 vs 72%, respectively, at two years.

Similarly, in a prospective multicenter randomized study with 334 stage lll and lV patients, in which our institution participated, Bernier et al [3] (Table 1) compared postoperative combined radiation-chemotherapy with postoperative radiation alone. The authors found a significantly increased 5-year progression-free survival and overall survival rates in favour to the combined arm, with 47 vs 36%, and 53 vs 40%, respectively.

### Comparison with postoperative conventional radiation (p3DCRT) data

Table 1 shows outcome data of the above mentioned three large prospective p3DCRT trials [3-5] and two smaller prospective single institution series [2,13], addressing the outcome following p3DCRT combined with chemotherapy. In high risk cohorts treated with p3DCRT without chemotherapy [3-5], highly concordant LRC rates of 70 and 72% were reported, with an increase of approximately 10% up to ~82%, when chemotherapy was added [3,4]. Our high risk pIMRT-chemotherapy cohort resulted in higher control rates regarding LRC, disease free survival and overall survival, respectively.

We stratified our patients according to the risk factors established and used by Ang et al [5] (see section 'methods', and Table 4). Cooper et al [4] defined 'high risk' as any or all of the following: invasion of >/= 2 lymph nodes, ECE, R1. Bernier et al [3] used ECE, R1, PNI, and vascular tumor embolism for definition of high risk situations, while Porceddu et al [13] considered the presence of ECE, positive or close margins (<5 mm), and loco-regional recurrence as high risk features. Bachauds' et als' [2] patient inclusion criterion was ECE.

### Treatment associated factors impacting on loco-regional control in pIMRT

#### a) Total treatment time (TTT)

Postoperative IMRT was performed using dose painting with SIB. SIB schedules with a slightly increased dose per fraction translate into a mild treatment acceleration. Treatment acceleration has been shown to improve outcome in historic 3DCRT series. The SIB schedules used in pIMRT are listed in Table 5. Mild treatment acceleration was therefore performed in 24 patients irradiated with doses per fraction higher than 2.0 Gy (2.11, 2.2 or 2.3 Gy/d) to the boost volume.

On the other hand, our collective showed unfavourable features regarding the treatment timing, with a surgery to start of radiation interval of > 6 weeks, and the TTT (time from surgery to the last radiation therapy day) of >12 weeks in the majority of patients. The TTT in the four patients who locally recurred was 12, 14, 18, and 22 weeks, respectively. Reasons for this unfavourably long TTT are multifactorial (such as time gap between surgery and referral for first presentation, dental care prior to planning computer tomography, IMRT planning time, and others more).

Both the interval between surgery and the start of radiation of > 6 weeks, and the TTT of >13 weeks, have been shown to translate into a highly significantly lower loco-regional control rate by Ang et al [5]. Consecutively, shortening of TTT may be a possibility to further optimize outcome.

#### b) concomitant radiation-chemotherapy

The advantage of the combined radio-chemotherapeutic approach in the postoperative situation has clearly been shown. The statistically significant increase of LRC is approximately 10% [2-4,13] (Table 1).

#### c) IMRT

IMRT technique offers a clear benefit in terms of increased treatment tolerance (xerostomia [14], mandible bone necrosis [8]), and may result in an increase of LRC in difficult anatomic situations (e.g. posterior pharyngeal wall [7], upper level 2, skull base) by permitting somewhat higher doses to the tumor. IMRT seems to be an additional beneficial factor in improving outcome in postoperative HNC. Better outcome compared to historic 3DCRT cohorts has been reported for definitive IMRT (dIMRT) in pharyngeal tumors [15,7,16].

There are presently only few reports on pIMRT (Table 2). In conclusion, pIMRT in HNC, as reported by other centres [9-12,16], has resulted in local control or LRC rates ranging between 81 and 95%, respectively, which is in concordance with our results. A direct comparison between the cited pIMRT series is, however, limited because the risk factors have not been indicated.

In a historic small p3DCRT series from our institution (S Maurer, dissertation, Radiation Oncology, University Hospital of Zurich, 1996) from the time interval when conventional fractionation (5× 1.8 Gy per week) was replaced by an accelerated boost schedule (see section 'patients' in the method's section), the first 18 accelerated patients showed an actuarial 2-year local control of ~82% vs 68% in 41 conventionally treated patients, with a minimal/maximal follow up time of 16/63 months each group. The absolute recurrence rate was 22 vs 49%, respectively. No concomitant chemotherapy was given at that time.

We found no published reports comparing intra-institutional historic p3DCRT to pIMRT results.

## Conclusion

Surgery followed by postoperative IMRT in patients with high risk for recurrence resulted in high loco-regional tumor control rates compared with large prospective 3DCRT trials.

## Methods

### Patients

Between January 2002 and August 2006, 71 of 320 HNC IMRT patients were treated with postoperative IMRT (pIMRT). During the first year, when IMRT was clinically implemented at our institution, for capacity reasons only few postoperative patients could be included into the IMRT program. Since the beginning of 2003, all HNC patients referred for curative (definitive or postoperative) radiation therapy have been treated with IMRT. No patient selection was performed. Patient and tumor characteristics are presented in Table 3.

In 9 of 15 patients without clinical or radiological signs of nodal involvement, no neck dissection has been performed.

Therapeutic decisions for these patients were made at weekly interdisciplinary HNC tumor boards. All patients were operated at the joint Head and Neck Surgery, or Head, Neck and Maxillofacial Surgery, both at the University Hospital of Zurich. Similarly, histopathologic examinations and diagnosis have been performed by head and neck tumor specialists at the Institution of Pathology at the University Hospital of Zurich.

During the course of irradiation, all patients were clinically assessed at regular weekly intervals, at 2 weeks and at 2 months after completion of treatment.

Approximately 6 weeks after completion of treatment all patients were also seen regularly in our joint clinics at the Department of Head and Neck Surgery or Head, Neck and Maxillofacial Surgery. Further follow up visits were scheduled every 2 – 3 months in the first 2 years, 3 – 4 monthly in the third year. When clinical and/or endoscopic examination showed no evidence of disease annual radiological investigations were performed. Suspect findings were specified with computed tomography (CT), magnetic resonance imaging (MIR), and/or positron emission tomography/CT (PET/CT), suspect lymph nodes by needle aspiration and/or biopsy, respectively.

Since 1991, we used a risk stratification following the scheme described by Peters et al [1], when the authors showed the prognostic significance of the risk factors as listed in Table 4. In patients with low risk of recurrence, usually no postoperative radiation was performed. Because the data of Peters et al showed a loco-regional recurrence rate of approximately 30% after 63–68.4 Gy (oral presentation at ESTRO 1990, Monte Catini), we changed the postoperative fractionation to a concomitant boost regime, otherwise we used a dosage comparable to the dose as used by Peters et al. This institutional dose concept for postoperative situations was basically taken over when IMRT as a novel technology was implemented (see section 'radiation' below).

Actuarial disease outcome was calculated using Kaplan Meier survival curves.

Results were compared with data from 3DCRT series and IMRT cohorts reported from other centres.

Risk levels are defined as follows, based on the pathologic risk factors listed in Table 4:

• low risk: no adverse pathologic factor

• intermediate risk: only one adverse factor other than extra-capsular extension

(ECE)

• high risk: ECE, or >/= 2 adverse factors

In addition, we considered recurrences as intermediate risk, and as high risk, when in combination with one of the adverse factors listed above.

### Radiation treatment

#### Schedules

Total doses and doses per fraction of the SIB-IMRT schedules used are listed in Table 5.

High risk patients were treated with mean 67 Gy (60–70), intermediate risk patients with mean 64 Gy (60–68), respectively. Prescribed doses were calculated as the mean of the nominal dose to the high dose planning target volume (PTV1).

One patient has been treated with 2.3 Gy per session to 62.1 Gy for bulky nodal disease.

#### Planning target volumes (PTVs)

All patients were treated using simultaneously integrated boost technique [6].

-high dose planning target volume (PTV1):

High risk regions (the area of operated large tumors, areas of operated tumors with positive resection margin (R1), operated lymph node metastases measuring >3 cm, nodes with ECE, multiple positive nodes) were included in the PTV1. The operated primary and nodal gross tumor volume (pGTV) has been defined by contouring the anatomic region of the initial primary and nodal GTV, considering the preoperative diagnostics and the clinical and histological findings. The PTV1 includes the pGTV with a safety margin of between 1.0 and 1.5 cm, and was extended also 1.0 to 1.5 cm above and below. All patients underwent a pre-therapeutic fused positron-emission tomography and computed tomography (PET-CT), facilitating the identification of initial gross tumor volumes.

-elective planning target volume (PTV2): for defining the elective lymph node regions, the RTOG standard atlas (RTOG homepage: ) has been used.

Regions with intermediate risk were treated with doses of 56–60 Gy, elective lymphatic pathways have been treated with 46–54 Gy, respectively.

Bilateral nodal irradiation has been performed in all tumors extending to the midline (except of nodally negative sinonasal cancer), in all large pT2 and any size >pT2 staged primaries. In cases of unilateral involvement or cN0, the upper part of level II was not included.

### Chemotherapy

All high risk patients with no specific contraindications were simultaneously treated with cisplatin based chemotherapy (n = 59, 83% of all pIMRT patients). One cisplatin application consisted of 40 mg/m2/radiation week. 5 patients had 1–3 applications, 54 patients tolerated 4–7 applications, respectively.

12 patients had no cisplatin chemotherapy; two of them underwent combined cetuximab therapy.

The high percentage of combined treated individuals is related to the fact that IMRT was implemented in our institution towards the end of 2001, when first data on the benefit of combining radiation with chemotherapy were available [3], confirming earlier results of Bachaud et al [2]. In consequence, the combined modality approach in a postoperative setting was adopted as our institutional standard.

## Competing interests

The author(s) declare that they have no competing interests.

## Authors' contributions

GS and CG designed the study. GS drafted the manuscript. KF collected and analysed the histopathologic risk feature data.

SS and RZ reviewed the manuscript and participated in collecting data of the analysed operated patients.

CG, BD and UL reviewed and corrected the manuscript. All authors read and approved the final manuscript.
